# A Multidisciplinary Approach to Congenitally Missing Central Incisors: A Case Report

**DOI:** 10.7759/cureus.21911

**Published:** 2022-02-04

**Authors:** Carlos Jurado, Nicholas G Fischer, Chin-Chuan Fu, Akimasa Tsujimoto

**Affiliations:** 1 Prosthodontics, Texas Tech University Health Sciences Center El Paso School of Dental Medicine, El Paso, USA; 2 Minnesota Dental Research Center for Biomaterials and Biomechanics, University of Minnesota, Minneapolis, USA; 3 Department of Restorative Dentistry, University of Alabama at Birmingham School of Dentistry, Birmingham, USA; 4 Department of Operative Dentistry, University of Iowa College of Dentistry, Iowa City, USA

**Keywords:** orthodontic, dental anomalies, incisor, lithium disilicate crown, crown lengthening, occlusion

## Abstract

Hypodontia is one of the most common developmental problems of human dentition. The treatment of missing maxillary central incisors is always a challenging task, often requiring a multidisciplinary approach. This case report describes such a multidisciplinary approach for a female patient with congenitally missing maxillary central incisors and class II division 1 occlusion. Significant horizontal overlap was present with class II division 1 occlusion in a patient with a history of cleft palate. Implant therapy was thereby not an option. Orthodontic treatment was provided to decrease the horizontal overlap and reposition the teeth. Esthetic crown lengthening was performed and monolithic lithium disilicate crowns were placed. Critical analysis of the treatment plan through cooperation among specialists is required to obtain the ideal result. Orthodontic treatment may be necessary to close or gain more space, followed by implant placement (if acceptable), and restorative treatment. It is important to create the treatment plan through a multidisciplinary approach involving orthodontists, surgeons, and restorative specialists before initiating treatment.

## Introduction

Tooth agenesis of permanent dentition is a common developmental anomaly appearing in approximately 1.9% to 9.6% of populations, excluding third molars [1]. Hypodontia, which is most common compared to oligodontia or anodontia, is the absence of less than 5 teeth (excluding third molars) whereas oligodontia is the absence of more than 6 teeth (again, excluding the third molars) [2]. Previous studies have shown that permanent dentition has been shown to be more commonly affected than primary dentition [3]. In general, studies have shown large differences in prevalence between different populations; for example, some populations have a prevalence under 2% [4]. Specifically, a higher prevalence has been reported in Europe and Australia compared to North America. Tooth agenesis may appear as part of a non-syndromic disorder or a syndrome in which agenesis happens as an isolated trait [5]. The most common missing teeth from agenesis are, in order of most commonly missing, third molars, second lower premolars, maxillary lateral incisors, upper second premolars, and, in some rare cases, the central incisors [6]. Although the etiology of agenesis remains unclear, inheritance of an autosomal dominant trait is suggested to be the most common among the many other causes.

Congenitally missing maxillary central incisor treatment presents many challenges to many clinicians. The first, and most critical, is the requirement of a coordinated multidisciplinary team-based approach involving many specialties including orthodontic, prosthodontic, and periodontal treatment to achieve ideal functional and esthetic results [7]. Diligent diagnosing and information gathering, with reliance on other specialists, as well as careful communication with the patient, is needed to formulate an optimal treatment plan to satisfy functional and esthetic outcomes. Factors that need to be considered include teeth alignment, space closure, occlusion, alveolar bone status, facial analysis, and periodontal condition. Additional factors that should be considered include parafunctional and personal habits of the patient. At last, any formulated treatment plan should be as least invasive option available.

The applicability of space closure often depends on the suitability of the maxillary permanent canines for modification in order to substitute for lateral incisors. A long-standing controversy, and a key treatment planning question, is whether to open or close spaces via prosthetic substitution or by mesial movement, respectively [8]. This becomes critically important because the goal of the multidisciplinary approach is to achieve a symmetrical appearance in the esthetic zone [9].

The purpose of this case report was to describe a multidisciplinary approach for a female patient with congenitally missing maxillary central incisors and class II division 1 occlusion.

## Case presentation

**Figure 1:**
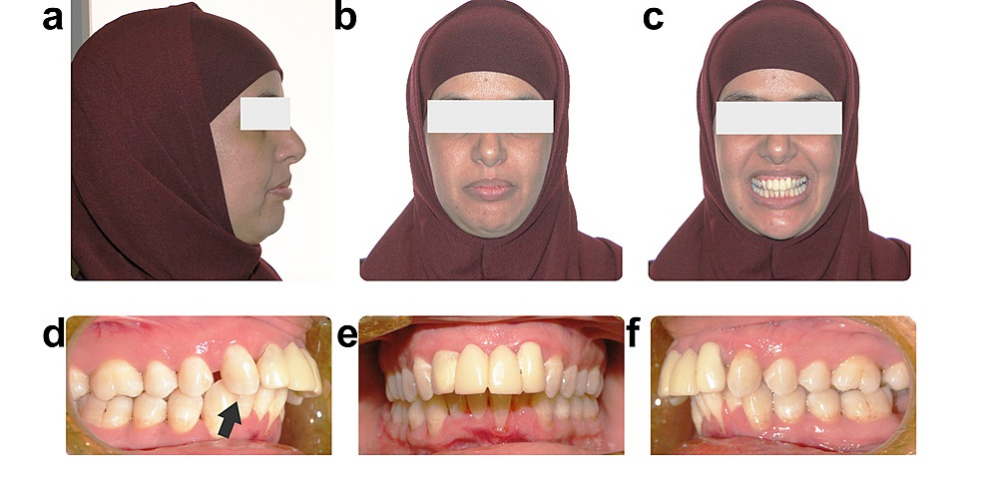
The image shows (a, b, and c) initial facial and (d, e, and f) intraoral photographs. Arrow indicates area of interest.

A 27-year-old female patient was referred with a chief complaint of dissatisfaction with the esthetic quality of her smile. Upon examination, the patient was diagnosed with congenitally missing central incisors. The missing central incisors had been previously restored with a fixed partial denture (FPD) from teeth #7-10 (maxillary lateral and central incisors) with abutments on teeth #7 and #10 (maxillary lateral incisors). Significant horizontal overlap was present with class II division 1 occlusion (Figure 1a-1f). The patient had a history of cleft palate which was repaired in childhood. As a result of the previous cleft palate, implant therapy was not an option, as was thoroughly explained to the patient. The dissatisfaction with the fixed partial denture influenced the patient's selection of treatment.

**Figure 2:**
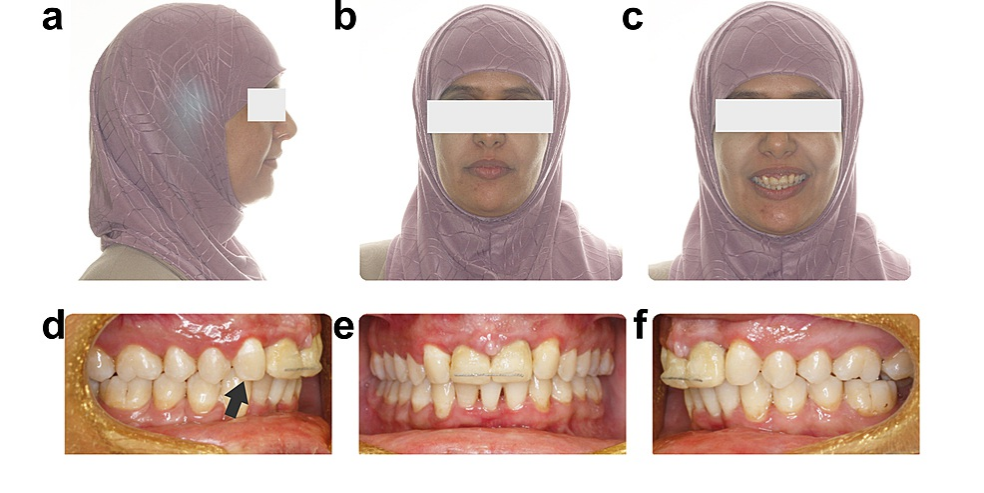
The image shows (a, b, and c) facial and (d, e, and f) intraoral photographs after orthodontic treatment. Arrow indicates the area of interest.

After evaluation and patient consent, diagnostic impressions were made to fabricate diagnostic casts. Pontic #8 and #9 (maxillary central incisors) were trimmed away from the maxillary diagnostic cast; abutment #7 and #10 were repositioned to central incisor positions to simulate the result after orthodontic movement. A diagnostic wax-up was then fabricated for the anterior region; #7 and #10 were waxed to simulate the size and morphology of central incisors to allow better evaluation of the space distribution for the orthodontist. Orthodontic treatment was then provided to decrease the horizontal overlap and reposition the teeth. The existing FPD (#7-10) was removed and provisional crowns for #7 and #10 were fabricated using polymethacrylate (Jet Acrylic, Lang Dental Manufacturing, Wheeling, IL) according to the wax-up. Both provisional crowns were cemented using resin-modified glass ionomer cement (RelyX Luting Plus, 3M Oral Care, St. Paul, MN) to endure the orthodontic movement. Orthodontic treatment was utilized to reposition the lateral incisors to the central incisors position, the canines to the lateral incisor positions, and the premolars to the canine positions. The teeth were also slightly palatalized in order to obtain Class I occlusion. The patient was instructed on standard methods to maintain her oral hygiene and decrease her caries risk during this two-year period of orthodontic therapy. The patient, prior to treatment, was aware of the timeline that orthodontic treatment would take. These instructions included oral hygiene instructions, two dental prophylaxes a year, dietary control of sucrose, and recommended the use of fluoride toothpaste (Figure 2a-2f).

**Figure 3:**
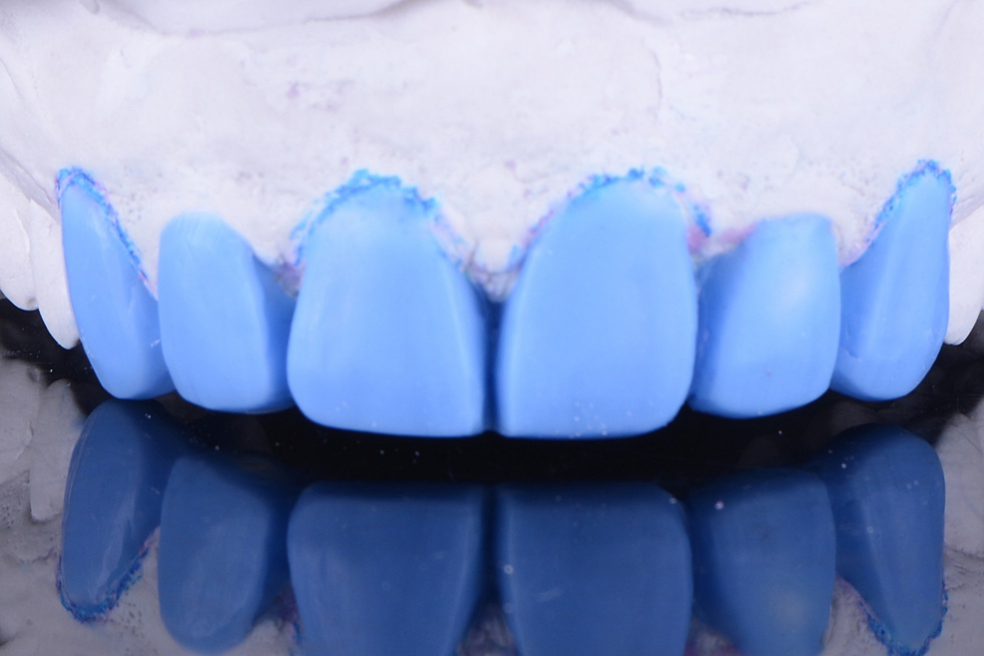
The image shows the diagnostic wax-up.

**Figure 4:**
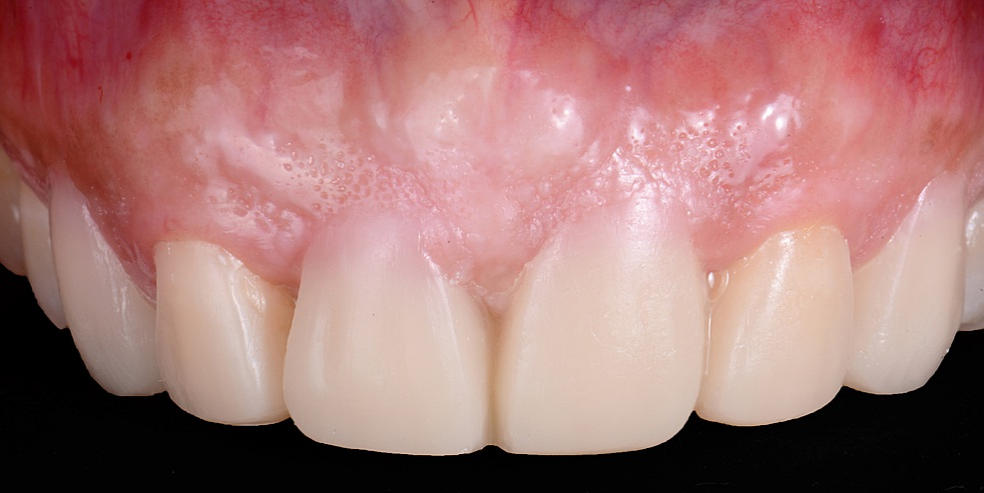
The image shows the intraoral mock-up.

After finishing orthodontic treatment, a diagnostic impression was taken using polyvinyl siloxane impression material (Virtual 380, Ivoclar Vivadent, Schaan, Liechtenstein). Diagnostic casts were fabricated in type IV stone (Fujirock, GC, Tokyo, Japan) and mounted using a face bow and a semi-adjustable articulator (Model 2340Q, Whip Mix, Louisville, KY). A diagnostic wax-up was performed and a silicone index was fabricated for clinical mock-up (Figure 3). The wax-up was transferred to the mouth using a bis-acryl provisional material (Integrity, Dentsply Sirona, York, PA) in the silicone index to evaluate the esthetics, phonetics, patient smile, occlusion, and overall patient comfort (Figure 4). The mock-up was modified and further used as a reference for tooth preparation and the crown lengthening procedure. Calibration grooves were made with different thickness diamond burs for minimal preparation while performing the tooth preparation. New provisional restorations for teeth #5-12 were fabricated using polymethacrylate resin (Jet Acrylic, Lang Dental Manufacturing, Wheeling, IL).

**Figure 5:**
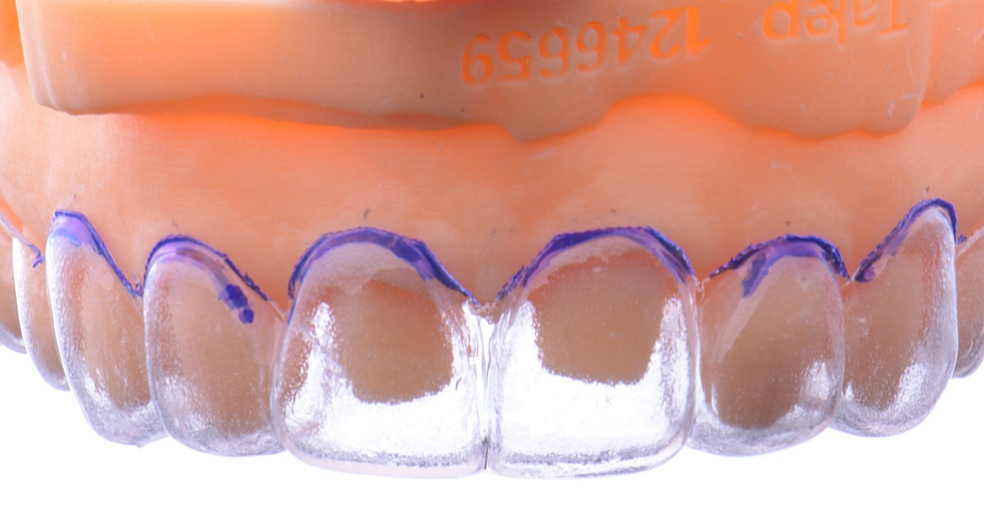
The image shows the fabrication of the crown-lengthening guide.

Subsequently, a 1.5mm thick clear crown-lengthening guide (Clear Splint Biocryl, Great Lakes Dental Technologies, Tonawanda, NY) was fabricated with a thermal forming machine (Biostar, Scheu-dental GmbH, Iserlohn, Germany), and the gingival contour of the guide was trimmed following the scallop of the previous diagnostic mock-up (Figure 5). Esthetic crown lengthening was performed from #5 to #12 (maxillary first pre-molars, canines, lateral incisors, and central incisors) in order to harmonize her gingival contours followed by four months of healing time until the new gingival tissue level was stable.

**Figure 6:**
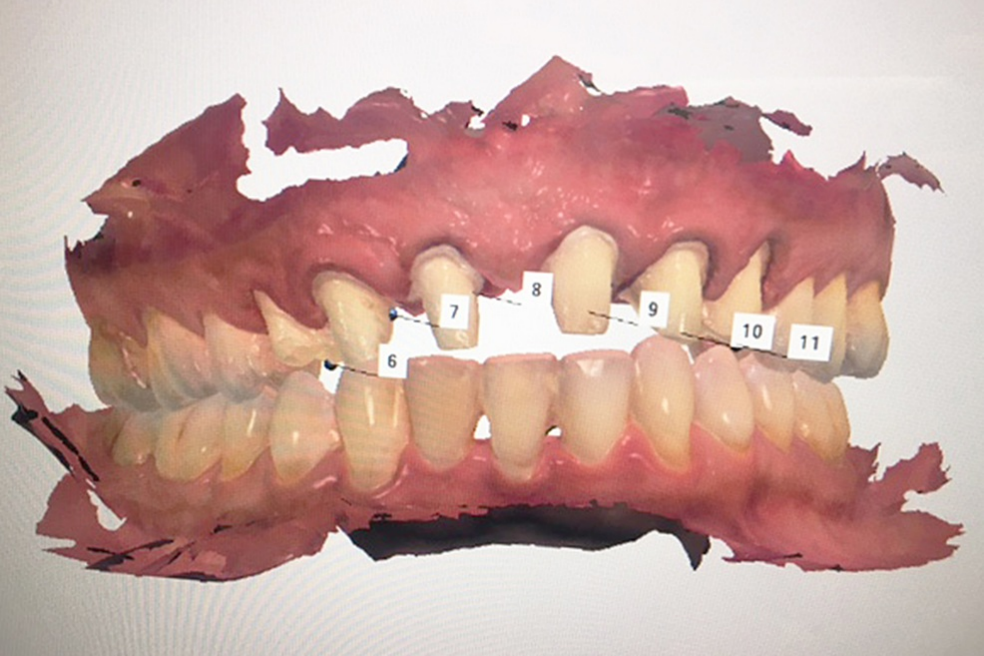
The image shows the digital impression.

After four months of healing, the tooth preparations were refined and the provisional restorations were relined. The final impression was taken using a digital scan (3Shape Trios, 3Shape A/S, Copenhagen, Denmark) (Figure 6).

**Figure 7:**
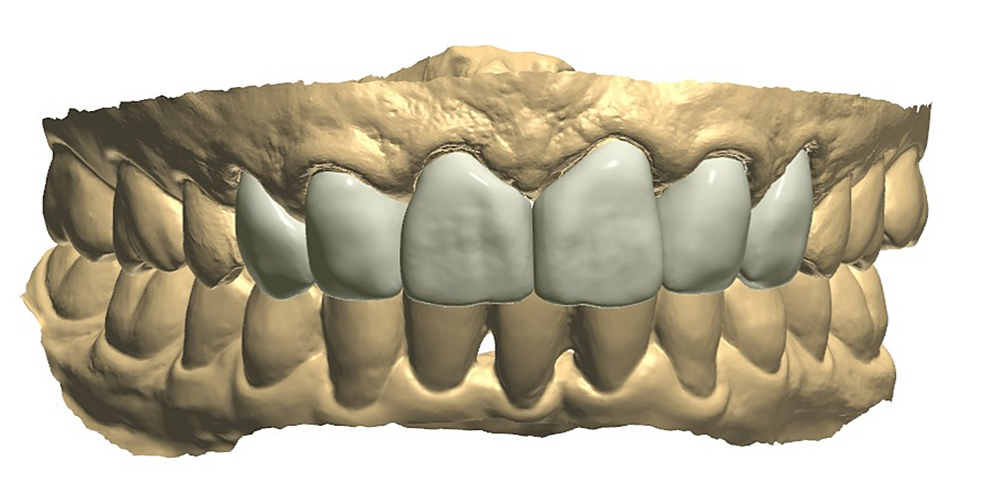
The image shows the design of the final restorations.

Final restorations were designed with the desired contours (Figure 7).

**Figure 8:**
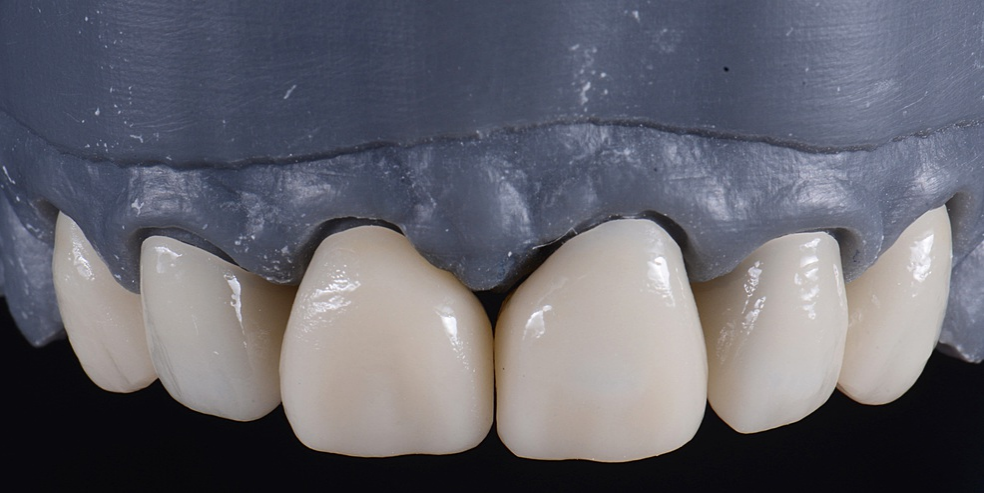
The image shows the fabrication of the final ceramic restorations.

Monolithic lithium disilicate crowns (IPS e.max CAD, Ivoclar Vivadent, Schaan, Liechtenstein) were fabricated (Figure 8).

**Figure 9:**
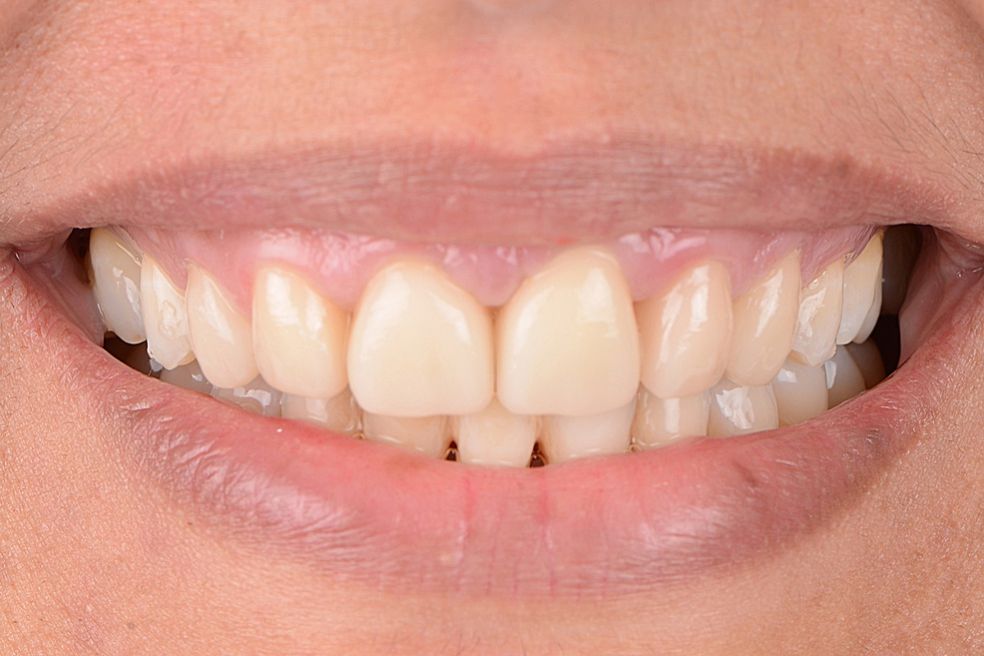
The image shows the final facial view.

The final restorative crowns were seated clinically, occlusal contact was verified, and protrusive and lateral excursive interferences were removed with a fine diamond bur and diamond polishers (Dialite LD, Brasseler, Savannah, GA). All crowns were then bonded using resin cement (RelyX Ultimate, 3M Oral Care, St. Paul, MN) (Figures 9).

**Figure 10:**
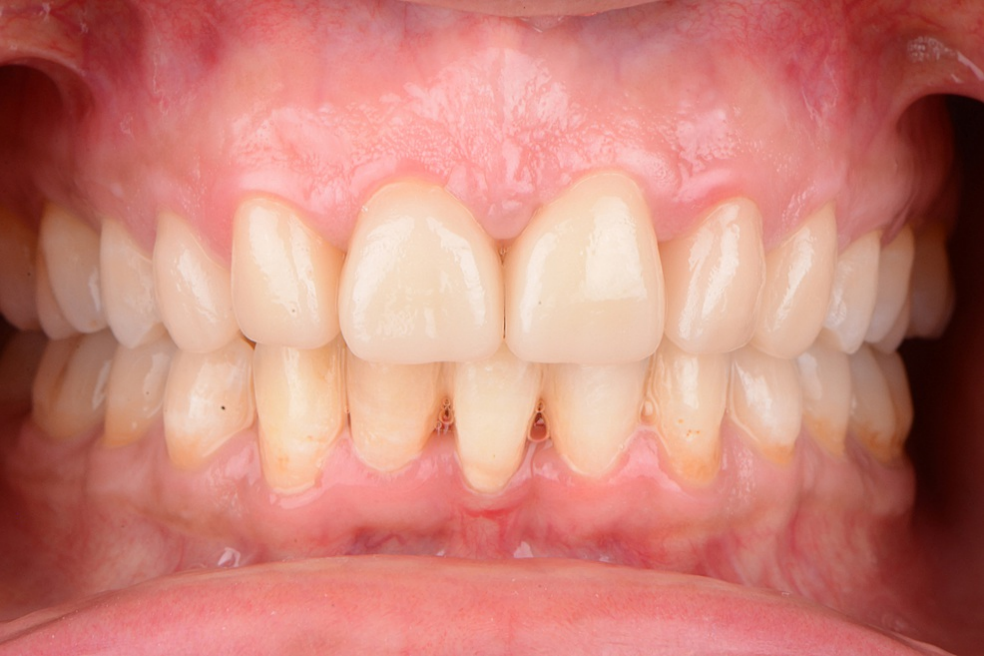
The image shows the five-year intra-oral follow-up. The golden ratio relationship between maxillary central incisor and lateral incisors was measured based on this image. Here, when considering the congenitally missing central incisors, we found the central incisors to be 1.645 times the width of the later incisors, which is close to the golden ratio of 1.6.

A new impression was taken and an orthodontic retainer was fabricated to secure the alignment of teeth. The patient was recalled every six months. No problems nor complications were noted before or at the five-year follow-up examination after discussion with the patient (Figure 10).

## Discussion

The overall treatment goal should be the maintenance of healthy oral structures while keeping original tissues using interventions that are simple, effective, predictable, and in harmony to the para-oral structures [10]. However, every single patient is different because clinical conditions inevitably change. However, a multidisciplinary analysis should, when used properly, almost always lead clinicians to an appropriate treatment resolution using minimally invasive dentistry techniques whenever possible. Here, the use of orthodontic treatment reduced the amount of tooth structure removed, which is an advantage. In particular, hard and soft tissues both need to be considered and treatments such as flapless extractions [11] followed by implant therapy, are good examples of minimally invasive dentistry [12-14]. Indeed, this case report is unique given the history of cleft palate in the patient and the inability to place implants. The inability to place implants in this patent was a disadvantage that was overcome.

Orthodontic treatment typically plays a key role in diagnosis and treatment of patients with congenitally missing teeth. However, adjunctive restorative care in conjunction with periodontal therapy is often necessary to re-create ideal gingival contours and teeth shape in the esthetic zone from canine to canine. Therefore, comprehensive treatment planning is necessary to achieve optimal final esthetics. Some have suggested the golden ratio as one outcome for optimal final esthetics. Following the idea of the golden ratio, if we assume that the width of the lateral incisor is 1.0 arbitrary units (au), then the central incisor would be 1.6 au, and the canine would be 0.6 au. Here, when considering the congenitally missing central incisors, we found the central incisors to be 1.645 au at five years follow-up, which suggests the golden ratio was achieved.

It is common for decisions regarding the ideal selection of restorative dental materials in complex multidisciplinary dental treatments to be difficult for each case. Composite resins are practical if small tooth modifications are needed [15]. On the other hand, indirect restorations using ceramics are good options if large shape and color modifications are necessary. Ceramics are indicated for restoring the anterior area because of their optimal esthetic properties, like optical effects such as texture, shade, and translucency [16]. Ceramics were strongly indicated here as this case involved gum operations and large-scale prosthetics.

Minimally invasive dentistry is important whenever adhesive bonding systems are used. Enamel plays a fundamental role in adhesive dentistry, so it is important to make use of less invasive preparation designs in order to preserve the maximum tooth structure [17]. Crown-lengthening guides are one way to take advantage of minimally invasive dentistry. Our learned experiences show that the orthodontist should not have a one-size-fits-all approach for all missing maxillary central patients but instead individualize considering diagnostic criteria for each individual patient and evaluate the positives and negatives of each of the different treatment possibilities [18]. The reality of costly orthodontics treatment, in addition to the cost of restorations needed, is a situation that sometimes presents itself for some patients. Indeed, while technological advances are incorporated into practice, the cost for patients need not be forgotten. The presence of other existing problems such as low-quality bone or aged restorations complicates the treatment of some patients. The long list of requirements to achieve an optimal treatment goal may be too much for the patient to go through [19]. The clinician must be prepared to present multiple treatment options and be receptive for feedback from the patient. Overall we emphasize that “evidence-based decision-making in the treatment of severe hypodontia is not yet feasible” [20] and a highly-specific treatment plan, considering all these factors, is necessary, as well as further research on outcomes for hypodontia.

Previous dental care received by the patient did not fulfill her esthetic demands causing the patient to look for a second opinion in order to receive more comprehensive care. The achieved outcome was clinically acceptable even though orthodontic treatment was initially provided without prosthodontics and periodontics consultation. However, the authors believe that a more comprehensive initial evaluation including prosthodontics and periodontics feedback could be more beneficial prior to orthodontic treatment. Patient education regarding maintenance and follow-up is also important to ensure satisfactory long-term outcomes.

## Conclusions

The overall treatment of congenitally missing central incisors requires a multidisciplinary approach from orthodontists, periodontists, and restorative dentists. We demonstrated this herein with a successful follow-up at five years in a case of congenitally missing maxillary central incisors and class II division 1 occlusion. With a careful diagnosis, creativity in treatment planning, collaboration between specialists, and the dedication of the patient, a challenging case was accomplished with a result that was pleasing to the patient.
